# Dataset on social demographic and employee job satisfaction in the Nigerian manufacturing company

**DOI:** 10.1016/j.dib.2018.04.143

**Published:** 2018-05-05

**Authors:** Adewale O. Osibanjo, Ebeguki E. Igbinoba, Hezekiah O. Falola, Kayode O. Awe

**Affiliations:** Covenant University, Ota, Nigeria

**Keywords:** Job satisfaction, Social demographic, Structural Equation Modeling

## Abstract

The dataset on the effects of social demographic on job satisfaction was obtained through self-administered questionnaire. The survey was situated in a Nigerian manufacturing company and the valid ninety two copies of the questionnaire were analyzed by AMOS 21. Structural Equation Modelling (SEM) analysis was carried out on the constructs. In addition, further analysis of the data will assist in establishing the significant level of demographic on job satisfaction.

**Specifications Table**

**Table:** 

Subject area	*Management*
More specific subject area	*Human Resources Management*
Type of data	*Primary data (tables and figures)*
How data was acquired	*Research instrument, SEM,*
Data format	*Raw, analyzed (SPSS & AMOS)*
Experimental factors	*The survey is based on data obtained from ninety two respondents of a Manufacturing company using SPSS and Structural Equation Modelling to identify the effects of social demographic on job satisfaction among the respondents studied.*
Experimental features	*Social demographic characteristics of respondents are essential in determining job satisfaction of employees*
Data source location	*Ogun, Nigeria*
Data accessibility	*Data as attached*

**Value of the data**

• The outcomes of the data can assist in managerial decisions such as recruitment and selection processes.
• The analyzed data can provide insights into the generational differences and how each affects job satisfaction.
• Managers can also leverage on the data for workforce diversity management.

## 1 Data

**Fig. 1:**
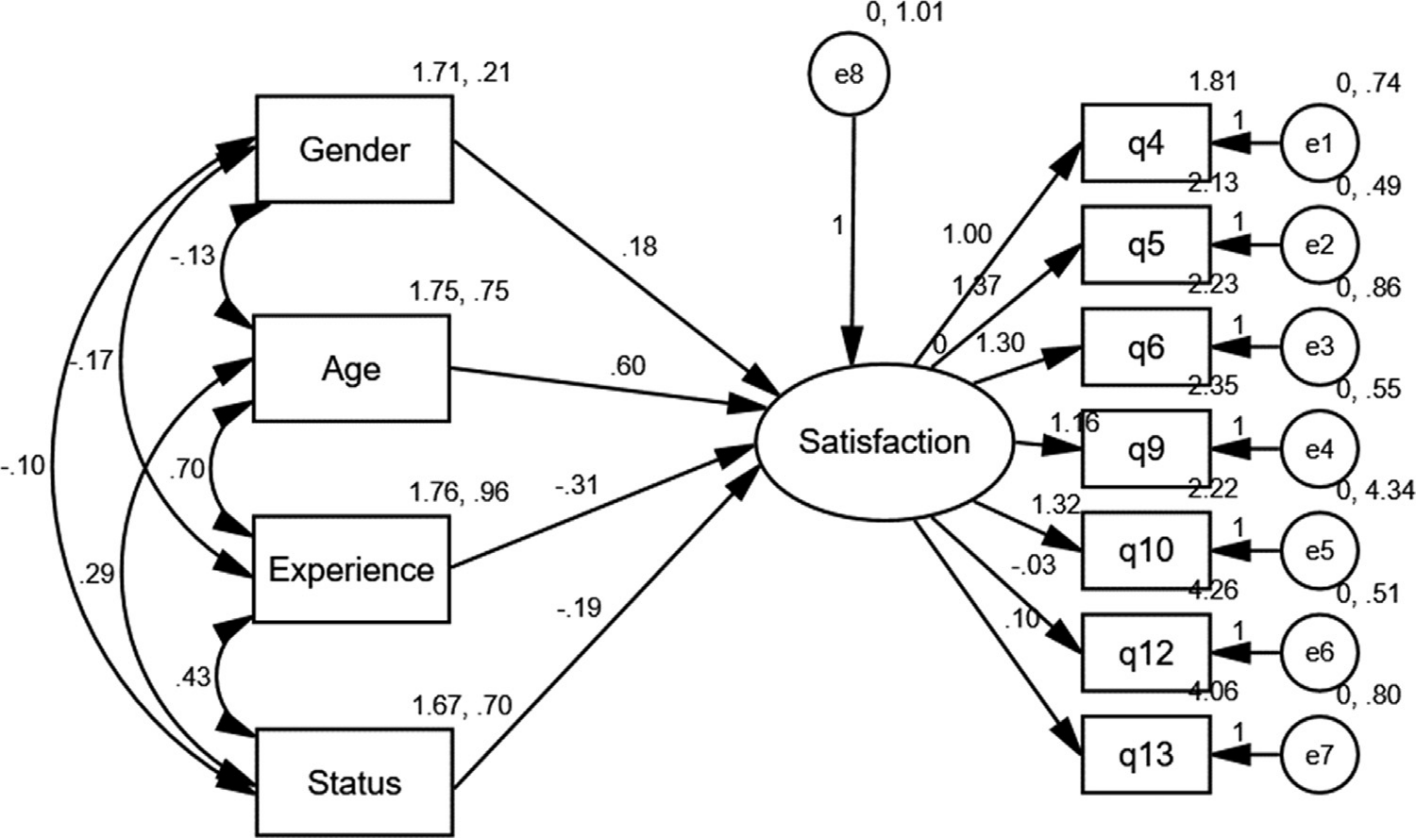
Social demographic and job satisfaction model.

The dataset contained effects of social demographic on job satisfaction. The survey is premised on quantitative method and the Structural Equation Modelling (SEM) statistical tool was adopted to identify the significant effects of demographic characteristics of employees of a manufacturing company on job satisfaction [1], [2]. The results of the analysis of the model as depicted in Fig. 1, also depicted in Table 1 is the demographic characteristics of the respondents. It is important to note that the data presented has academic and managerial implications. For instance if the data is analysis, it will help the management of manufacturing industry to have deep understanding into the significant role of social demographic characteristics in enhancing job satisfaction. Similarly, management of the manufacturing industry may leverage on the data for the purpose of decision making. In a related development, other researchers can make use of the data for further investigation on the subject matter. Meanwhile, both the management and employees of the sampled organisation were adequately informed about the objective of the study and the permission was granted to administer the research instrument. In addition, respondents were equally assured of the confidentiality of their responses.

**Table 1:** Demographic characteristics.

**Social characteristics**		**Percentage (N92)**
**Gender**	Male	29.3%
	Female	70.7%
**Age**	21–30 years	47.8%
	31–40 years	34.8%
	41–50 years	12.0%
	51years and above	5.4%
**Marital status**	Single	52.2%
	Married	32.6%
	Divorced	10.9%
	Widow	4.4%
**Work experience**	0–5 years	53.3%
	6–10 years	27.2%
	11–15 years	9.8%
	15 years and above	9.8%

## 2 Experimental design, materials and methods

The statistics presented in this data set was based on the quantitative study conducted that examined the influence of social demographic variables on job satisfaction in a manufacturing company in Nigeria. Descriptive survey research design which help to assess sample at the specific time without inconsistencies was adopted. One of the leading manufacturing firms in Ogun State, Nigeria was sampled. The study population consisted all employees of the sampled manufacturing firm. Researchers used complete enumeration of employees because the population of the study is relatively small. Data was collected with the use of a structured questionnaire. However, Structural Equation Modeling (AMOS 22) was used for the analysis of data [3], [4], [5]. The analysis of this data would give an in-depth understanding of what the management of the manufacturing firms and other stakeholders should do to effectively manage workforce diversity thereby enhancing job satisfaction.

## References

[1] Osibanjo O.A., Salau O.P., Falola H.O. (2014). Modeling the relationship between motivating factors; employee' retention; and job satisfaction in the Nigerian banking industry. J. Manag. Policies Pract..

[2] Osibanjo A.O., Salau O.P., Falola H.O., Oyewunmi A.E. (2016). Workplace stress: implications for organizational performance in a Nigerian Public University. Bus.: Theory Pract..

[3] Ibidunni O.S., Osibanjo O.A., Adeniji A.A., Salau O.P., Falola H.O. (2016). Retention and organizational performance: a competitive positioning in Nigerian banking sector. Period. Polytech. Soc. Manag. Sci..

[4] Bentler P.M. (1990). Comparative fit indexes in structural models. Psychol. Bull..

[5] Hu L.T., Bentler P.M. (1999). Cutoff criteria for fit indexes in covariance structure analysis: conventional criteria versus new alternatives. Struct. Equ. Model..

