# Supplementary material for: Dataset on social demographic and employee job satisfaction in the Nigerian manufacturing company
Source: Data Brief. 2018 May 5;19:13–5. doi: 10.1016/j.dib.2018.04.143 (PMC5992971; doi:10.1016/j.dib.2018.04.143)
Supplement: Supplementary file 1 — Transparency document [file mmc1.doc]

**Falola Hezekiah Olubusayo (Ph.D)**

Department of Business Management

College of Business and Social Sciences,

Covenant University, Ota, Ogun State, Nigeria

[hezekiah.falola@covenantuniversity.edu.ng](mailto:hezekiah.falola@covenantuniversity.edu.ng)

+234 703 5518 559

**April 5, 2017**

The Editor,

Data In Brief

Dear Sir,

**DECLARATION OF CONFLICT OF INTEREST**

I, Dr. Falola H.O and my colleagues write to declare that there is no conflict of interest traceable to our data paper “Dataset on Social demographic and Employee Job satisfaction in the Nigerian Manufacturing Company”

”

”

Thank you.

Yours faithfully,


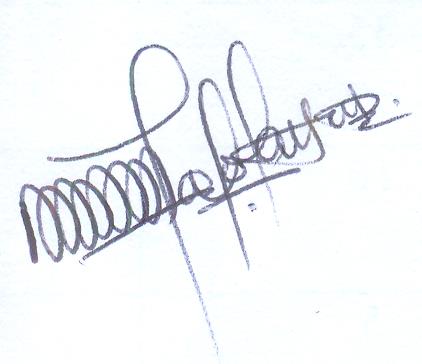


FALOLA H.O (PhD)

**Corresponding Author**
